# Sorption of selected antiparasitics in soils and sediments

**DOI:** 10.1186/s12302-021-00513-y

**Published:** 2021-07-02

**Authors:** Andre Patrick Heinrich, Timm Zöltzer, Leonard Böhm, Manuel Wohde, Sara Jaddoudi, Yassine El Maataoui, Abdelmalek Dahchour, Rolf-Alexander Düring

**Affiliations:** 1 Institute of Soil Science and Soil Conservation, Research Center for Biosystems, Land Use and Nutrition (iFZ), Justus Liebig University Giessen, Giessen, Germany; 2 Laboratory of Materials, Nanotechnology and Environment (LMNE), Faculty of Sciences, Mohammed V University in Rabat, Av Ibn Battouta Agdal, BP1014 Rabat, Morocco; 3 Département Des Sciences Fondamentales Et Appliquées, Institut Agronomique Et Véterinaire Hassan II, Rabat, Morocco

**Keywords:** Sorption, Pharmaceuticals, Environmental fate, Environmental distribution, *K*_D_, *K*_OC_, Moxidectin, Avermectin, Desorption, Africa

## Abstract

**Background:**

Veterinary pharmaceuticals can enter the environment when excreted after application and burden terrestrial and aquatic ecosystems. However, knowledge about the basic process of sorption in soils and sediments is limited, complicating regulatory decisions. Therefore, batch equilibrium studies were conducted for the widely used antiparasitics abamectin, doramectin, ivermectin, and moxidectin to add to the assessment of their environmental fate.

**Results:**

We examined 20 soil samples and six sediments from Germany and Morocco. Analysis was based on HPLC-fluorescence detection after derivatization. For soils, this resulted in distribution coefficients *K*_D_ of 38–642 mL/g for abamectin, doramectin, and ivermectin. Moxidectin displayed *K*_D_ between 166 and 3123 mL/g. Normalized to soil organic carbon, log *K*_OC_ coefficients were 3.63, 3.93, 4.12, and 4.74 mL/g, respectively, revealing high affinity to organic matter of soils and sediments. Within sediments, distribution resulted in higher log *K*_OC_ of 4.03, 4.13, 4.61, and 4.97 mL/g for the four substances. This emphasizes the diverse nature of organic matter in both environmental media. The results also confirm a newly reported log K_OW_ for ivermectin which is higher than longstanding assumptions. Linear sorption models facilitate comparison with other studies and help establish universal distribution coefficients for the environmental risk assessment of veterinary antiparasitics.

**Conclusions:**

Since environmental exposure affects soils and sediments, future sorption studies should aim to include both matrices to review these essential pharmaceuticals and mitigate environmental risks from their use. The addition of soils and sediments from the African continent (Morocco) touches upon possible broader applications of ivermectin for human use. Especially for ivermectin and moxidectin, strong sorption further indicates high hydrophobicity and provides initial concern for potential aquatic or terrestrial ecotoxicological effects such as bioaccumulation. Our derived *K*_OW_ estimates also urge to re-assess this important regulatory parameter with contemporary techniques for all four substances.

**Graphic abstract:**

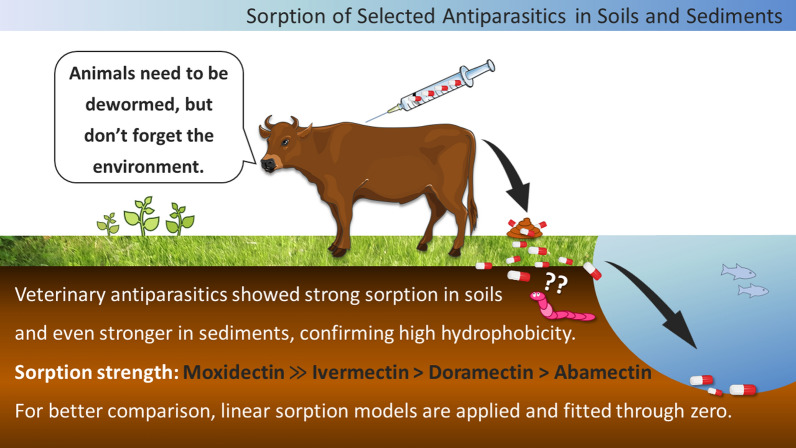

**Supplementary Information:**

The online version contains supplementary material available at 10.1186/s12302-021-00513-y.

## Background

Discovery of the anthelmintic, actinomycete-derived macrocyclic lactones in the 1970s and their advancement into widely available antiparasitic agents came as nothing less than a medical and economic revelation [1, 2]. Grouped into avermectins and milbemycins, the efficient broad-spectrum endectocides for humans and animals revolutionized treatment of parasitic infestations [3]. Hailed as the ‘wonder drug from Japan’ [4], the avermectin derivative ivermectin was added to the World Health Organization model list of essential medicines [5]. A valued antiparasitic and safe for human use, ivermectin is also considered as a new malaria vector control tool [6, 7]. Almost unparalleled in its benefits for human health [8], ivermectin (IVM) was initially developed as a veterinary drug. Similar macrocyclic lactones include the avermectins abamectin (ABA), doramectin (DOR), and eprinomectin (EPR) as well as moxidectin (MOX), a milbemycin agent [3].

The use of pharmaceuticals for animals and humans can be accompanied by the release of drug residues into many environmental compartments. Particularly veterinary medicinal products (VMPs) for livestock, poultry or aquaculture come with the risk of direct drug excretion onto agricultural soils, involuntary application via manure fertilization, and release via runoff or erosion into surface waters. Exposure routes also include drug manufacturing and disposal, and all exposure scenarios raise the question of potential ecotoxicological effects and environmental fate of VMPs [9]. In this regard, sorption of VMPs and contaminants in soils and sediments is a fundamental process which governs the interdependence of fate, bioavailability, and ecotoxicity of a substance [10, 11]. Monitored by the European Medicines Agency (EMA), VMPs set to be registered in the European Union (EU) must undergo environmental risk assessments [12, 13]. Unfavorable, in this context, for avermectins and MOX is that animals excrete them largely unmetabolized, mainly within days after application, and primarily bound to feces. This feature is ascribed to their hydrophobic nature and an active excretion process via P-glycoprotein [14, 15]. Despite their extensive use as VMPs, only a limited number of sorption studies exist for macrocyclic lactones. In contrast, hundreds of soil sorption observations are available for major plant protection products, such as atrazine [16]. Reflecting the medical significance of IVM, most studies investigating soil sorption focus on this drug [17–19]. Others investigate ABA [20], EPR [21, 22], or multiple agents at once [23]. To varying degrees, the overarching observation is the tendency of these substances to strongly bind to soil organic matter. This is indicated by a high organic carbon–water partition coefficient (*K*_OC_). However, with sorption as a fundamental process in soil chemistry [24], the data situation on the fate of these VMPs seems insufficient.

Another complication for environmental risk assessments of the four antiparasitics is the lack of reliable and transparent octanol–water partition coefficients (*K*_OW_). A routinely assumed order of hydrophobicity (as log *K*_OW_) appears to be: IVM (3.2 [25], presumably used in a marketing request [26]), ABA (4.0 [27] to 4.4 [28]), DOR (4.4 [29]), MOX (4.77 [30], presumably referred to by the EMA [31]). However, the reported methodology behind these values can be deficient or absent. This is reiterated by the EMA [32] which also cites 4.4 as log *K*_OW_ for DOR, but addresses the inappropriately used shake-flask method. This methodology is also stated for the 4.4 value of ABA [28] and for MOX [30]. Furthermore, a report funded by the German Environment Agency on environmental properties of antiparasitics, compiled by Römbke et al. [13], concluded that a log *K*_OW_ of 3.22 underestimates this key hydrophobicity indicator for IVM. While this value by Halley et al. [25] is cited frequently [17, 33, 34], the 2019 report implicates at least a 240-fold increase in hydrophobicity when expressed as log *K*_OW_. In 1989, a method to determine the log *K*_OW_ of potentially highly hydrophobic substances like IVM had not been standardized. It was only introduced in 2006 by the Organisation for Economic Co-operation and Development (OECD) with guideline 123 [35]. Compared to the shake-flask method, this slow-stirring technique is considered more reliable for highly hydrophobic substances [12]. The technique was applied by the Fraunhofer-Institute for Molecular Biology and Applied Ecology (Schmallenberg, Germany), yielding a new log *K*_OW_ of 5.6 (± 0.3) for IVM [13]. This assessment is backed by curated data from the U.S. Environmental Protection Agency predicting a median log *K*_OW_ of 5.41 for IVM’s main component IVM B_1a_ [36].

From a regulatory perspective, a log *K*_OW_  >  4 for VMPs indicates a potential for bioaccumulation to occur in the environment, although multiple criteria need to be considered [12, 37]. In this context, the EMA’s Committee for Medicinal Products for Veterinary Use concluded that MOX-containing VMPs for cattle, sheep, and horses might harbor persistent, bioaccumulative, and toxic properties [38]. Fabrega and Carapeto [39] compiled that as a result of environmental concerns, 20 referral procedures of VMPs have been triggered to re-assess environmental risks post-authorization. Six of these products were antiparasitics. It is noteworthy that the European Union is committed to identify knowledge gaps and to address potential environmental risks of pharmaceutical residues and investigate their fate [40].

### Non-target effects of macrocyclic lactones

Extensive reviews by Liebig et al. [33], Lumaret et al. [41], Finch et al. [42], and Junco et al. [43] summarize environmental risks accompanying the unintentional release of macrocyclic lactones and are cause for concern. Acute and chronic effects are observed especially for coprophagous species. Though well documented, knowledge about fate and toxic effects of these drugs on non-target organisms is ever-evolving. Beyond dung and soil, aquatic biota can also be harmed if antiparasitics enter surface waters and sediments [44–46].

### How would antiparasitics end up in sediments?

Compelling evidence for this pathway is presented in a field study by Mesa et al. [47] who treated cow herds with IVM and monitored drug concentrations in the wetlands used for grazing. IVM was detected in manure, water, sediment, and macrophytes as well as in wetland invertebrates and fish. Environmental IVM loads increased with animal count and injection frequency. For DOR, Kumirska et al. [48] reported field-concentrations in water, sediment, and fish at a sampled river, with DOR in water exceeding predicted no effect concentrations for *Daphnia magna*. Since ABA is also used as a pesticide, runoff or erosion from treated fields can enter adjacent water bodies [49, 50], enabling transport into sediments. Discharge of antiparasitics into water and sediment, besides direct excretion or transfer from fields, may also be relevant in aquaculture. There, concerns for environmental exposure have been raised for IVM [51], ABA [52], and the ABA derivative emamectin benzoate [53]. When used to control sea lice infestations, IVM can be quantified in low concentrations in marine sediments around fish farms [54]. In water, DT_50_-values of  <  6 [55] and 15.9 h [56] have been reported for IVM in simulated sediment/water systems. This indicates rapid dissipation from aqueous media; presumably binding onto suspended particles and sediment. However, there are no known studies documenting the sorption of macrocyclic lactones in sediments. At the same time, wetlands and sediments are invaluable nurseries for benthic and hyporheic invertebrates as well as emergent aquatic insects (e.g., *Ephemeroptera*, *Plecoptera*, *Trichoptera*) which carry nutrients and biomass to terrestrial habitats [57, 58]. The drivers of global insect decline are under discussion [59] and it is worth investigating to what extent environmental chemicals and pharmaceuticals may contribute. Although sediments can act as both sinks and sources for contaminants and serve vital functions in aquatic food chains, environmental risk assessment in this compartment is fragmentary [34]. Diepens et al. [60] reiterate this current underrepresentation in regulatory frameworks. If risks of antiparasitics or other VMPs are to be assessed, environmental risk assessment begins with meaningful exposure assessment including a substance’s fate in all plausible environmental compartments. Thus, we aim to establish comparable sorption data for antiparasitics in soils and sediments which provide a basis for regulatory decisions.

### Experimental approach

We investigated the sorption of 4 macrocyclic lactones used as antiparasitic VMPs: the avermectins ABA (also used as pesticide), DOR, IVM, and the milbemycin MOX. Sorbates were used simultaneously in each sorption experiment and could be determined at once within an analytical run. The methodology for sorption studies is standardized in OECD guideline 106 [61] to predict substance partitioning in soils. As a novelty, we also performed sorption experiments with six sediments in addition to 20 investigated soil samples. Sorption of these drugs in sediments has not been reported before. We also present, to our knowledge, first-time data from a batch equilibrium study on the sorption of these VMPs in soils and sediments from the African continent.

This work promotes linear modeling with constrained intercepts to derive comparable sorption coefficients that enable robust regulatory decisions. To assess the general hydrophobicity of the antiparasitics and validate our sorption results, we derive and review *K*_OW_ estimates from *K*_OC_ coefficients.

## Materials and methods

### Soil and sediment samples

German soil samples (label DE) were provided by the Hessian Agency for Nature Conservation, Environment and Geology (HLNUG). From a pool of samples, 17 were selected for sorption studies. The selection was based on OECD guidance instructions [61]. These samples represent a range of physicochemical properties, soil horizons, textures, sampling depths as well as pasture and crop locations throughout the state of Hesse. Moroccan samples (label MA) were taken in the Gharb Basin region in the northwest of Morocco with a soil auger, collecting the top 20 cm of soil and sediment. Crop residues on soils were omitted since fields were previously cultivated for various cereals. Bed sediments were sampled along Sebou River (Oued Sebou; MA07 to MA09) and Loukkos river (Oued Loukos; MA04–MA06) with MA04 closest to the Atlantic coast at Merja Zerga lagoon. Before sampling, sediments were cleared of debris. Distance to shore or embankment was 1.5–2 m to sample sediments that were continuously underwater. Table 1 shows the physicochemical properties of soil and sediment samples. In contrast to German samples, the Moroccan samples represent a Mediterranean climate. They are characterized by generally higher pH values in the carbonate buffer range, resulting from limestone and marl limestone deposits in the basin [62]. Samples were air-dried and sieved to 2 mm. Water content was determined by drying aliquots at 105 °C.

**Table 1:** Physicochemical properties, origins, and sampling depths of soils and sediments for the sorption experiments. Soils labeled DE were taken in Germany; samples labeled MA originated in Morocco

Label	Site	Depth (cm)	%OC^a^	C/N^b^	pH^c^	CEC^d^	Reference soil group^e^	Texture (% w/w)		
								Sand	Silt	Clay
DE01	Crop	90–120	0.08	3.7	6	19.2	Luvisol (siltic)	2	61.7	36.3
DE02	Crop	0–20	5.9	20.9	7.4	19.8	Regic anthrosol	59	24.7	16.3
DE03	Crop	65–90	0.73	50.2	7.6	23.2	Terric anthrosol (stagnic)	12.2	26	61.9
DE04	Crop	40–100	0.14	5.6	5.5	4.3	Cambisol (loamic)	79.4	16.4	4.2
DE05	Crop	60–90	0.15	4.8	6.3	18.9	Luvisol (siltic)	2.1	63.4	34.5
DE06	Crop	80–120	0.11	2.7	6.4	11.9	Planosol	12.9	54.5	32.7
DE07	Crop	40–60	0.29	5.7	7.4	32.5	Cambisol (clayic)	5.3	33.4	61.2
DE08	Pasture	30–55	1	6.9	6.8	22.4	Vertic cambisol	3.1	49	47.9
DE09	Crop	0–30	1.8	11.3	5.9	9.9	Umbrisol	51.8	36.3	11.9
DE10	Pasture	30–80	0.83	7.5	6	14.7	Gleyic cambisol (siltic)	13.5	62.5	24
DE11	Pasture	0–30	2.72	8.9	5.3	27.7	Vertisol	4.5	65.9	29.5
DE12	Pasture	0–5	3.15	8.9	4.4	19.9	Umbrisol (loamic)	30.8	50.2	19
DE13	Pasture	0–25	3.57	8.9	5	23.9	Stagnic gleyic cambisol	4.4	62.8	32.8
DE14	Pasture	0–5	3.89	9.7	4.6	23.5	Umbrisol (siltic, leptic)	28.9	49.1	22
DE15	Crop	0–15	6.01	17.5	6.9	27.5	Terric anthrosol (stagnic)	53.3	26.1	20.7
DE16	Crop	95–100	0.9	68.2	7.7	21.5	Terric anthrosol (stagnic)	10.6	27	62.5
DE17	Pasture	0–10	4.7	8.9	5.5	32.8	Gleysol	21.1	52.2	26.6
MA01	Crop	0–20	2.09	18.6	7.4	n/d	Vertic cambisol	10.2	49.7	40.1
MA02	Crop	0–20	1.93	16.7	7.6	n/d	Vertisol	4.3	28.1	67.6
MA03	Crop	0–20	1.33	18.2	7.6	n/d	Vertisol	1.7	27.8	70.6
MA04	Sediment	0–20	0.43	–^f^	7.7	n/d	Not applicable	95.7	1.7	2.6
MA05	Sediment	0–20	1.23	12.5	7.7	n/d	Not applicable	2.2	35.3	62.5
MA06	Sediment	0–20	0.42	26.3	7.6	n/d	Not applicable	60.2	18.9	20.9
MA07	Sediment	0–20	1.62	19.7	7.7	n/d	Not applicable	3.1	32.5	64.4
MA08	Sediment	0–20	1.38	30.1	7.5	n/d	Not applicable	17.5	41.4	41.1
MA09	Sediment	0–20	0.62	50.7	7.5	n/d	Not applicable	19.5	53.4	27.1

*n/d* not determined

^a^Weight percentage of soil/sediment organic carbon, following DIN ISO 10694

^b^Carbon–to–nitrogen ratio

^c^pH measured in a solution of 0.01 mol/L CaCl_2_; following DIN ISO 10390

^d^Potential cation exchange capacity in cmolc/kg; following DIN ISO 13536

^e^Reference soil groups according to the World Reference Base for Soil Resources [63] were derived using the German Soil Survey Guidelines, 5th ed. (KA5) and field data from HLNUG. Moroccan soils were characterized on-site. Sediment classification [64] is not provided since gravel content was not available

^f^Not available, minimal N content made determination impossible

### Materials

**Fig. 1:**
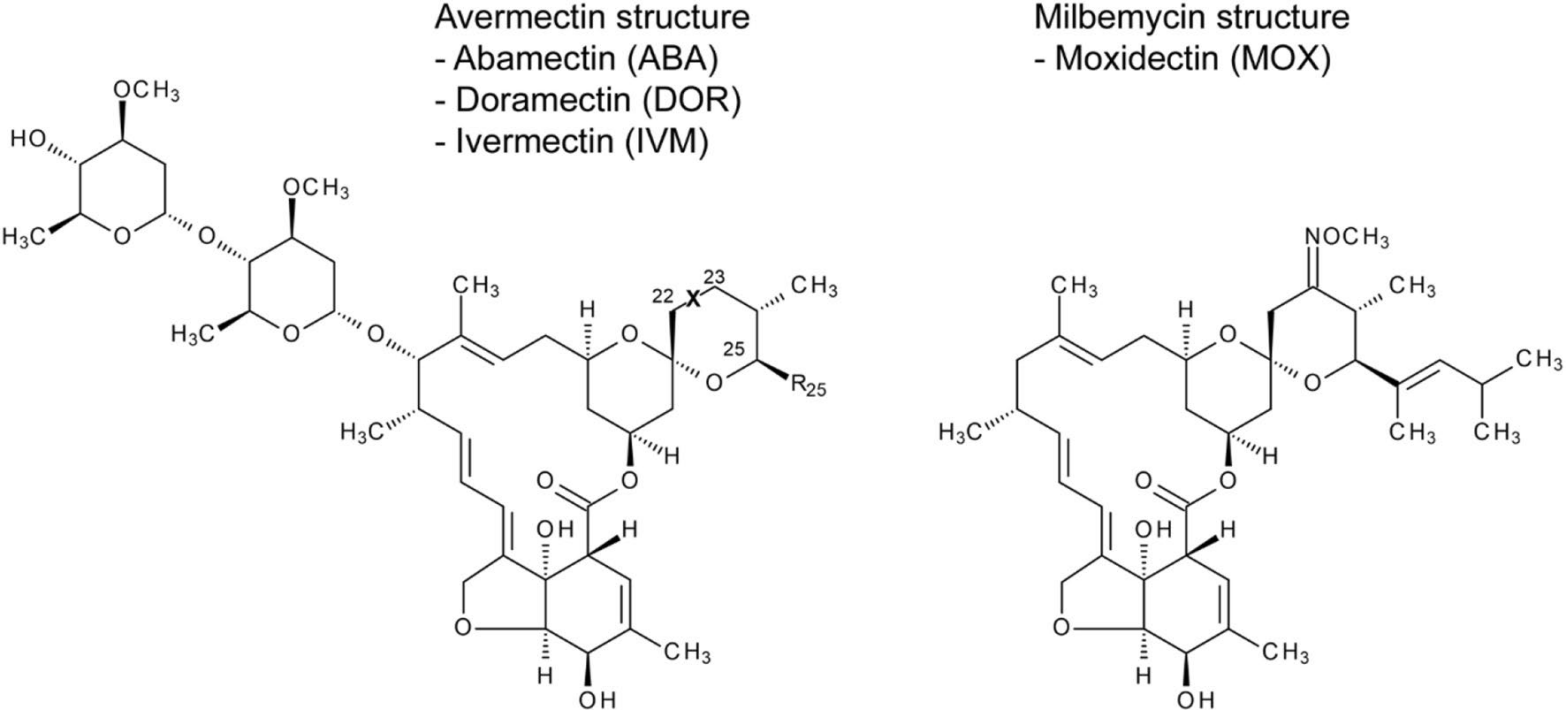
Chemical structures of the examined antiparasitics. Differences for avermectins (left) are: abamectin (ABA): X  =  double bond, R_25_  =  CH(CH_3_)CH_2_CH_3_ and CH(CH_3_)_2_; doramectin (DOR): X  =  double bond, R_25_  =  cyclohexyl group; ivermectin (IVM): X  =  single bond, R_25_  =  CH(CH_3_)CH_2_CH_3_ and CH(CH_3_)_2_. The chemically related milbemycin moxidectin (MOX) is shown on the right. Own illustration based on Shoop et al. [3]

ABA and IVM are mixtures of semisynthetic avermectin B_1_ derivatives. They contain at least 80% B_1a_ component (C-25 s-butyl group) and less than 20% B_1b_ component (C-25 isopropyl group), while DOR holds a sole cyclohexyl group at C-25. Chemically related, the smaller MOX molecule is a semisynthetic derivative of the milbemycin nemadectin, a fermentation product of *Streptomyces cyanogriseus*, whereas avermectins are derived from *Streptomyces avermitilis* [3, 65]. Structural differences are shown in Fig. 1.

Antiparasitics were purchased as analytical standards (CAS-no.; product-no., supplier; purity) in powder form: ABA (71751-41-2; 31732-100MG, Sigma-Aldrich; 98.6%), DOR (117704-25-3; DRE-C13083000, LGC Standards; 96.0%), IVM (70288-86-7; DRE-CA14488000, LGC Standards; 96.0%), and MOX (113507-06-5; DRE-CA15335000, LGC-Standards; 94.6%). Acetonitrile (ACN) (20060.320) and propan-2-ol (84881.320P), both  ≥  99.9% purity, came from VWR International; Calcium chloride dihydrate (102382) from Merck. Derivatization chemicals (> 99.0% purity) were bought at Sigma-Aldrich: N-methylimidazole (336092), triethylamine (T0886), trifluoroacetic anhydride (106232), and trifluoroacetic acid (302031). Purified water was prepared with a Milli-Q^®^ system. We used a CHROMABOND^®^ solid-phase extraction (SPE) system (MACHEREY–NAGEL), custom handblown 45 mL glass centrifuge vials with a PTFE-coated silicon seal inside the screw cap, 500 mg Strata C18-E SPE cartridges (8B-S001-HCL, Phenomenex), and 0.45 µm PTFE membrane syringe filters (WIC 79145, WICOM).

### Sorption experiments

Experiments were conducted according to OECD guideline 106 [61]. We suspended 1 g dried soil or sediment with 30 mL 0.01 mol/L CaCl_2_ solution in purified water for a 1:30 (w/v) solid/solution ratio. This ratio was elaborated in own preliminary studies and is situated between the ratios of 1:20–:40 (w/v) applied in a comparable study by Rath et al. [23]. Before spiking, solid samples were pre-shaken in the CaCl_2_ solution for 24 h. Since these antiparasitics represent highly hydrophobic substances, powdered analytical standards were dissolved in ACN to create stock solutions of 4 × 10^6^ µg/L. These solutions were combined in equal proportions for a mixed solution containing 1 × 10^6^ µg/L of each substance. This was diluted into working solutions to simultaneously spike all drugs in a consistent volume of 30 µL ACN for a 0.1% (v/v) solvent concentration [61]. Sorption in soils DE01 to DE06 was not studied for MOX. In the ongoing sorption study series, we created the following test concentrations in the aqueous phase: 100, 200, 300, 400, and 500 µg/L (samples DE07–DE17); 100, 200, 300, 500, and 1000 µg/L (MA01–MA09); 500, 1000, 1500, 2000, and 2500 µg/L (DE01–DE06). After spiking, solutions were shaken for 48 h (sorption equilibrium time) while glass vials were wrapped with aluminum foil to prevent possible photodegradation. Controls contained soil/sediment samples with CaCl_2_ solution or CaCl_2_ solution spiked with antiparasitics absent of soil/sediment. An experimental duration of 48 h was selected to reach apparent sorption equilibrium between macrocyclic lactone concentrations in soil/sediment and the aqueous phase. This was based on own preliminary kinetic studies and is supported by comparable experiments. [17, 20]. An exemplary desorption assessment was performed after 72 h and is briefly addressed in the discussion. Systems were equilibrated using a horizontal lab shaker (KS-10 Swip, Edmund Bühler GmbH) at 250 rpm. German samples were spiked in duplicates, Moroccan samples in triplicates. All steps were performed under ambient laboratory conditions at 21 ± 1 °C.

### Sample processing

Analytical procedures were based on Wohde et al. [56, 66] and adapted as follows: SPE cartridges were conditioned with 10 mL propan-2-ol followed by 10 mL of a 1:3 mixture (v/v) of propan-2-ol and purified water. At 48 h shaking time, samples were centrifuged at 2820*g* for 30 min and 25 mL supernatant were added to a reservoir atop the SPE cartridges along with 8.333 mL propan-2-ol and 25 µL triethylamine. Dried cartridges were eluted with 10 mL propan-2-ol. Eluates were evaporated to dryness under an N_2_ stream at 60 °C. For reconstitution, 1000 µL ACN were added to each vial. Vials were then sonicated for 15 min, horizontally shaken (250 rpm) for 30 min, and again sonicated for 15 min. Between each step, samples were vortexed for 30 s. Subsequently, samples were derivatized and quantified by HPLC-fluorescence detection on an Agilent 1200 HPLC system as elaborated by Wohde et al. [66]. This was applied for all four test substances with 40 µL injection volume and a shorter gradient elution. Mobile phases were A (purified water) and B (ACN); flow 0.3 mL/min; gradient 0–10 min, 88–100% B; 10–11 min, 100% B; 11–20 min 100–88% B. Since a broad range of sample characteristics and expected sorption was covered in the overall study series, we used different linear calibration sets of mixed standard solution with at least seven calibration standards per individual calibration series. All calibration curves displayed a linear response with *R*^2^  >  0.998.

### Deriving distribution coefficients

**Fig. 2:**
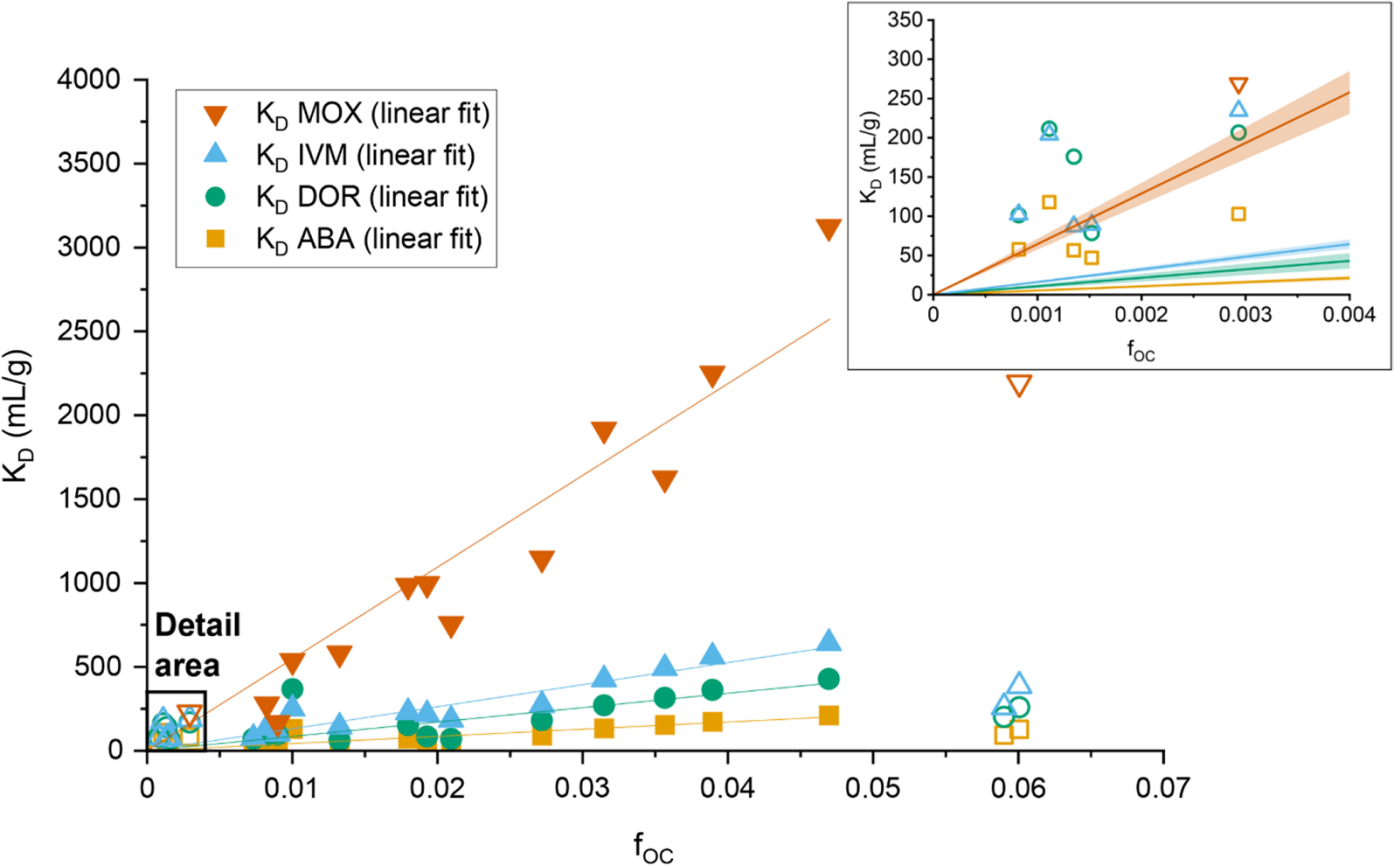
Experimentally determined *K*_D_ values of 20 soil samples from Germany and Morocco plotted against *f*_OC_ (organic carbon fraction) of each soil. Symbols, regression lines (and 95% confidence bands in the detail area, top right) depict results for abamectin (ABA), doramectin (DOR), ivermectin (IVM), and moxidectin (MOX). Hollow symbols of the same shape and color represent *K*_D_ values that were not included in *K*_OC_ calculations. See Additional file 1 for all *K*_D_

**Fig. 3:**
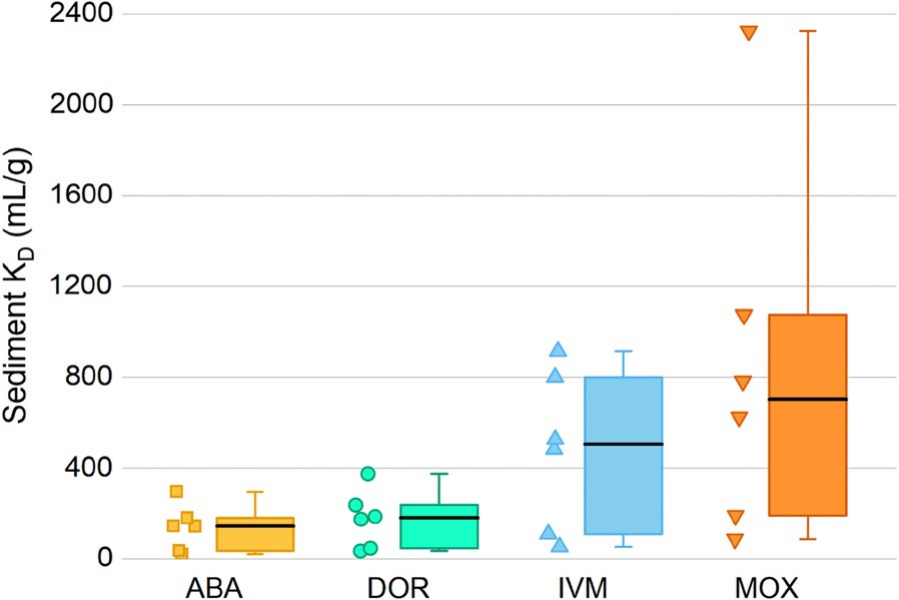
*K*_D_ results of six Moroccan sediments with individual values alongside boxplots. Boxes stretch from 25 to 75th percentile with whiskers showing minimum and maximum *K*_D_. The black line within each box is the median. See Additional file 1 for all *K*_D_

Soil and sediment samples were evaluated alike. Evaluation followed OECD guideline 106 [61]. The distribution coefficient *K*_D_ is defined as the ratio of substance concentration in the solid-phase *C*_s_(eq) and the substance concentration in the aqueous-phase *C*_aq_(eq) at equilibrium with the equation:

1 $$ K_{{\text{D}}}  = \frac{{C_{{\text{s}}} \left( {{\text{eq}}} \right)}}{{C_{{{\text{aq}}}} \left( {{\text{eq}}} \right)}}, $$

where *C*_s_(eq) is expressed in µg/g, *C*_aq_(eq) in µg/mL, and the *K*_D_ in mL/g. The measured *C*_aq_(eq) was then used to indirectly estimate the remaining amount of substance in the solid phase, delivering *C*_s_(eq). While Eq. (1) holds true for a single set of a solid and an aqueous phase, we derived the *K*_D_ for each sample by plotting all concentrations and replicates. We obtained the *K*_D_ as the slope of a linear regression with the y-intercept constrained. The decision of constraining the y-intercept was deliberate and relied on Chappell et al. [67] who concluded that only if consistency was imposed on a set of linear equations, distribution coefficients could be compared among different soils which is an aim of this work. While different concepts exist to describe distribution with sorption isotherms, such as linear models or nonlinear approaches with the Freundlich and Langmuir equation, they remain of theoretical nature. Linear models assume proportional increase of sorbed amounts with increasing adsorbate concentration in the aqueous phase. They consider no competition of solutes which is a relevant aspect when investigating four substances at once [24]. A constant slope reflects that sorbates have much higher affinity for sorbents than for the aqueous phase. This benefits low and environmentally relevant concentrations and Rao and Jessup [10] suggest the use of linear isotherms if agricultural applications or pathways are considered. Nonlinear sorption isotherms from studies with five test concentrations [61] may appear insufficient to produce a reliable, steady intercept that is not overstated. Especially the lowest concentration step can entail the most uncertainty and could strongly affect the intercept. Organic carbon (OC) is considered largely responsible for sorptive properties in soils [68]. Thus, the *K*_D_ is normalized to this parameter to derive the *K*_*OC*_ in mL/g. The *K*_OC_ can serve as a tool to estimate the mobility of a chemical in soil [69] and is derived with the equation:

2 $$ K_{{{\text{OC}}}}  = \frac{{K_{{\text{D}}} }}{{f_{{{\text{OC}}}} }}, $$

where *f*_OC_ is the OC fraction of the soil/sediment [70] expressed as weight percentage of soil/sediment OC (%OC). Here, *f*_OC_ was chosen over %OC to directly plot *f*_OC_ vs. *K*_D_ values and derive the *K*_OC_ of multiple soils as slope of a linear regression. Both Freundlich and Langmuir models can make determining a tangible *K*_OC_ impractical. Further, as recommended in OECD guideline 106, we excluded soil samples with  <  0.3% OC from *K*_OC_ calculations for which we selected 13 (12 for MOX) out of 20. Figures 2, 3 are calculated and created using OriginPro 2020b (OriginLab Corporation, Northampton, MA, USA).

### Method validation

Limit of detection (LOD) and limit of quantification (LOQ) for the HPLC-method were estimated according to recommendations in guideline Q2(R1) by the International Council for Harmonisation of Technical Requirements for Pharmaceuticals for Human Use [71]. Based on the five lowest calibration standards (2, 5, 10, 50, and 100 µg/L), we used the calibration curve slope (*m*) and standard deviation (*σ*) of the response expressed as standard error of the y estimate (derived with the STEYX function in Microsoft Excel 2019). LOD is expressed as 3.3 × σ/m and LOQ as 10 × *σ*/*m*. These results are given in Table 2. Further shown is the total number of replicates from all batch studies (soils and sediments) which were above the LOQ. Control samples did not reveal irregularities in terms of analyte losses or cross-contamination nor relevant sorption to surfaces of laboratory equipment. Pre-empting the results section, the concentration range in which substances were found across all samples reveals a hierarchy in their tendency to remain in the aqueous phase. The trend in lowest *C*_aq_(eq) in µg/L in any replicate was ABA (1.2), DOR (0.49), IVM (0.31). Conversely, the order for highest *C*_aq_(eq) was IVM (785), DOR (812), ABA (986). MOX conflicts this trend (0.62–247 µg/L), but was not used in soils with low %OC where a high *C*_aq_(eq) is suspected.

**Table 2:** Limit of detection (LOD) and limit of quantification (LOQ) for antiparasitics in sampled aqueous phases of sorption studies using area response of 2, 5, 10, 50, and 100 µg/L calibration steps in *n*  =  3 measurements with *R*^2^. Analyte concentration enrichment during sample processing is considered

	Corresponding concentration in the sampled aqueous phase C_aq_(eq)			Samples above LOQ (%)
	LOD (µg/L)	LOQ (µg/L)	Mean *R*^2^	
ABA	0.53	1.61	0.996	100
DOR	0.56	1.71	0.995	100
IVM	0.55	1.66	0.996	99.0
MOX	0.67	2.02	0.993	99.2

We monitored the stability of fluorescent ABA, DOR, IVM, and MOX derivates for 20 min, 24, 48, and 72 h after derivatization (*n*  =  6). After a slight decrease over time, 72 h average fluorescence recovery remained at 86.5, 85.1, 92.7, and 89.3% for ABA, DOR, IVM, and MOX derivates compared to 20 min. Consistently, samples were measured within 24 h after derivatization. Measurements of up to 72 h after derivatization of calibration standards and samples should not impair overall results.

Although the HPLC protocol yields favorable separation, a quality control was performed. Chromatograms of standard solutions containing only a single analyte showed minor fluorescence at retention times other than the main peak. This is presumably attributed to the purity (94.6–98.6%) of purchased standards. IVM and MOX peaks showed no overlap with impurities of other analytes. We found a fluorescence increase for ABA and DOR main peaks of 1.1 and 3.4% and downscaled these accordingly. ABA and IVM sorption results will represent their major (> 96%) B_1a_ component.

For additional method validation, a standard soil (LUFA 2.2) was purchased from LUFA Speyer (loamy sand; 1.61% OC; 0.18% nitrogen; pH 5.6 (0.01 mol/L CaCl_2_); CEC 8.5 meq/100 g). Therewith, we performed a mass balance determination [61] at 300 µg/L spiked concentration. Liquid phase extraction was performed with the presented SPE method. Soils and vessel walls were extracted two times with 5 mL ACN. The overall recovery of spiked antiparasitics ranged from 86 to 118% for the four substances. Mean recoveries (± SD, *n*  =  4) were: ABA 90.8 (4.1), DOR 107 (4.1), IVM 112 (2.1), and MOX 115.9% (1.6). This indicates that there is no relevant degradation of analytes within 48 h of shaking. Test substances can be considered to be stable. Determination with the indirect method [61] should thus be appropriate. This is in line with previous mass balance and stability reports on the sorption of avermectins [19–21]. The same soil and drug concentration were used to compare sorption of all four analytes with sorption when only IVM is added. IVM slope *K*_D_ (± SE, *n*  =  4) was 532 (12) with only IVM and 471 (26) mL/g with four analytes. Under these conditions only negligible competition in sorption is indicated when all four substances are spiked at once. Additional information on the method validation is provided in the Additional file 1.

## Results and discussion

### Sorption in soils

Batch equilibrium studies were evaluated with linear sorption isotherms which served for calculation of *K*_D_ values. Plotting soil *K*_D_ against *f*_OC_ resulted in Fig. 2. It shows the *K*_OC_ as the slope of the linear regression through 13 (12 for MOX) selected soils with the intercept constrained which creates a narrowing confidence corridor. For soils included in this regression, K_D_ values ranged from 38 to 211 (ABA), 63 to 428 (DOR), 76 to 642 (IVM), and 166 to 3123 mL/g (MOX). This dispersion characterizes the variability of the selected soils. Individual K_D_ values are listed in the Additional file 1.

The detail area of Fig. 2 shows soils with less than 0.3% OC. The linear regressions with 95% confidence bands of the used dataset fall below the individual *K*_D_ values for each substance in this region of low *f*_OC_. Recommended in OECD guideline 106, the procedure of removing these soils from *K*_OC_ calculations is corroborated by Krahe et al. [72] who also showed that below 0.3% soil OC linear model approaches become uncertain. They argue that at low concentrations, the accuracy of OC analyses could be reduced and that other sorption surfaces could become more relevant. It appears reasonable to assume that with little OC available, substances more likely sorbed to other available surfaces like clay, silt or dissolved organic matter, therefore displaying a K_D_ above the regression. Of the thus five excluded soils, four displayed above average clay or silt concentrations among all 20 soils which could explain their relatively high sorption. Only DE04 with the highest sand content and low cation exchange capacity contradicts this rationale. With the presented parameters, its sorption behavior remains elusive. Both DE15 and DE02 showed relatively low *K*_D_ values and contradicted the linear trend. This could be attributed to the fact that both samples originated from soil horizons with only moderately decomposed organic material and DE15 displayed stagnic properties. However, quality and detailed composition of organic material and its influence on the pH would have to be considered. Less decomposed organic material can harbor more hydrophilic groups, indicated by a lower elemental H/O ratio [73]. More available hydrophilic groups can explain lower sorption for hydrophobic substances if OC is the main sorbent. Thus, we further excluded soils DE15 and DE02 with OC contents of 6.0 and 5.9% and in total seven out of 20 soils for the K_OC_ calculation which are shown with hollow symbols. In a meta-analysis on atrazine sorption, Ou et al. [16] concluded that soils with OC  >  6% should be considered as outliers.

Next, Table 3 displays derived cumulated *K*_OC_ values using the previously selected 13 soils. We distinguish between the preferable description as slope of a linear regression through the origin (RTO) and an ordinary least squares (OLS) regression with floating intercept. The table further shows *K*_OC_ values expressed as the mean and median of individual *K*_OC_ values from soils considered suitable for the linearized approach. This illustrates the ambiguity that comes with the need to define a single value which quantifies a substance’s sorption behavior.

**Table 3:** Summarized *K*_OC_ data for the selected soils (*n*  =  13 for ABA, DOR, IVM; *n*  =  12 for MOX) showing a linearized and averaged approach to define a cumulated soil *K*_OC_. All values in mL/g

Substance	Linearized *K*_OC_ approach^a^		Averaged *K*_OC_ approach		
	RTO *K*_OC_ (SE)	OLS *K*_OC_ (SE)	Mean (SD)^b^	Median	Range (min–max *K*_OC_)
ABA	4286 (319)	3769 (651)	4941 (2581)	4343	2653–13,032
DOR	8574 (1025)	7470 (2134)	10,133 (8334)	8866	3423–36,683
IVM	13,139 (611)	13,441 (1288)	13,266 (4137)	12,795	8850–25,109
MOX	54,721 (3136)	66,506 (5666)	47,046 (13,356)	48,555	18,493–66,522

^a^Expressed as slope of a linear regression of *K*_D_ vs. *f*_OC_ (±  standard error of the regression slope) with the y-intercept forced through zero (*RTO* linear regression through the origin) or floating (*OLS* ordinary least squares)

^b^Arithmetic mean with standard deviation (SD)

Linearized RTO log *K*_OC_ were 3.63 (ABA), 3.93 (DOR), 4.12 (IVM), and 4.74 mL/g (MOX). For the OLS model, the log *K*_OC_ were 3.58, 3.87, 4.13, and 4.82 mL/g, respectively. The OLS y-intercepts amounted to 15.1 (ABA), 32.2 (DOR),  − 8.8 (IVM), and  − 349.9 mL/g (MOX). This partially reflects the increasing steepness of sorption from ABA  <  DOR  <  IVM  <<  MOX. However, the negative y-intercepts for IVM and MOX may also illustrate the shortcomings of an OLS regression with a floating intercept since negative sorption at zero or minimal OC would be implausible.

The *R*^2^ for the RTO *K*_OC_ were 0.94 (ABA), 0.85 (DOR), 0.97 (IVM), and 0.97 (MOX). However, since a constrained y-intercept skews *R*^2^ calculations it makes it less meaningful and complicates comparison with *R*^2^ obtained from OLS. Instead, the standard error (SE) of both regressions can be an alternative measure [74]. In this regard, the RTO *K*_OC_ appears to provide a more suitable fit. The *R*^2^ of the OLS *K*_OC_ were 0.75 (ABA), 0.53 (DOR), 0.91 (IVM), and 0.93 (MOX). This reflects the wide spread of individual *K*_D_ values, especially for ABA and DOR. Thus, it is conceivable that sorption of the more hydrophobic IVM and MOX is better explained with the *K*_OC_ concept than for the slightly less hydrophobic ABA and DOR. This deduction is reiterated by Tolls [11] for hydrophobic VMPs and their soil interactions in general. For the core range of 0.3–4.7% OC in soils, the relation between OC and distribution is well explained for IVM and MOX. This range also broadly represents the %OC found in most European agricultural topsoils [75]. In low OC environments, other surfaces such as clay are more relevant, while especially with a higher %OC, organic matter quality and composition appear to skew the K_D_-f_OC_ relation. Lastly, we applied a Box–Cox transformation on all soil *K*_D_ values to ensure normal distribution and subjected them to a multiple linear regression with OriginPro 2020b to compare them to soil properties from Table 1. With *α * =  5%, soil OC demonstrated significant influence on ABA, DOR, and IVM *K*_D_. C/N and pH were significant predictors for ABA and DOR *K*_D_. The complete output is listed in the Additional file 1. While OC is a convenient and established estimator for contaminant sorption in soils, it is plausible that, together with the pH, the detailed composition of organic matter would also predict sorption in soils once a large enough number of samples is studied.

### Broader context of soil sorption

Litskas et al. [22] stressed that avermectin sorption in soils determines bioavailability for non-target organisms. They suspected that once incorporated into soil, avermectins could withstand degradation and possibly accumulate if microbial activity was reduced due to unfavorable abiotic conditions or biocides [76]. This could be true for soils where agriculturally used biocides or disinfectants are spread with manure, potentially combined with antibiotics, antiparasitics, or other VMPs. Occurrence and transformation of biocides in manure and their fate in soils are only marginally investigated [77, 78]. Moreover, biocide release into the environment could increase due to the SARS-CoV-2 pandemic [79].

For IVM, promising mass drug administrations to livestock to target malaria vectors [80] may increase drug release onto soils. If this approach is complemented with human IVM treatments [7, 81], aquatic pathways in sewage systems could be subject to monitoring and analysis. This makes thorough drug exposure and fate assessments necessary. And it signals the need to include soils from the African continent and other previously neglected regions into sorption studies to provide most-needed One-Health solutions. To realize a safe and sustainable agricultural production, revised herd management strategies may also provide ecological and economic benefits while reducing stress on dung arthropod communities [82]. A sophisticated proposal for post-authorization monitoring of antiparasitics already exists [83] and a deliberate drug use could further address emerging anthelmintic resistances [84].

Lastly, the observed sorption in soils is in line with reported distribution coefficients for ABA of *K*_D_ 10–161 mL/g [85] and for IVM of *K*_D_ 57–396 mL/g [17]. Rath et al. [19] described IVM sorption *K*_D_ between 60 and 1953 mL/g and desorption *K*_D_ between 47 and 431 mL/g. Previous ABA *K*_OC_ ranged from 1495 to 7893 mL/g [28]. For IVM, *K*_OC_ between 4000 and 25,800 mL/g were documented [17] and for MOX between 18,000 and 41,000 mL/g [30]. However, it is difficult to compare *K*_D_ data from linearly modeled sorption experiments with other studies which used Freundlich sorption isotherms to produce a *K*_F_. Although nonlinear models can provide a better fit, they lack comparability, especially when the Freundlich exponent differs significantly from 1. Based on Rath et al. [19] we performed a desorption experiment using the LUFA 2.2 standard soil by replacing the analyzed liquid phase with the same amount of fresh CaCl_2_ solution and shaking for 72 h. Mean percentual desorption (±  SD, *n*  =  6) at a single concentration amounted to: ABA 4.6 (0.3), DOR 3.5 (0.1), IVM 2.9 (0.1), and MOX 2.6 (0.1)%. While only a fragmentary approximation for a full desorption study [61], these percentages compliment the sorption data of the four antiparasitics in soils and indicate mostly irreversible sorption processes.

### Sorption in sediments

Compared to soils, sediment *K*_D_ showed a range from 21 to 296 (ABA), 35 to 376 (DOR), 53 to 915 (IVM), and 87 to 2326 mL/g (MOX). Less indicative, mean sediment *K*_D_ (mL/g) were higher for ABA (106 vs. 98) and IVM (394 vs. 287) but lower for DOR (137 vs. 197) and MOX (861 vs. 1196). The distribution of all sediment *K*_D_ results is illustrated in Fig. 3 which again reveals the strongest sorptive behavior by MOX.

Although sediment MA06 had the lowest %OC (0.42), it displayed the highest *K*_D_ values for ABA, DOR, and IVM, and the third-highest for MOX among the six sediments. Since it cannot be inferred from the limited sediment sample size if this behavior is an outlier or part of an unknown trend, we removed MA06 from further calculations. However, parameters other than %OC could be more relevant for sorption in MA06. In comparison, MA04 with the highest sand content and similarly low %OC expectedly proved to be least prone for sorption. Because sediments also had a lower average %OC than soils, the resulting log *K*_OC_ of 4.03 (ABA), 4.13 (DOR), 4.61 (IVM), and 4.97 (MOX) mL/g were higher compared to soils when using an RTO. As was observed in soils, sediment log *K*_OC_ also ranked MOX  >> IVM  >  DOR  >  ABA, again reflecting the diverging behavior of MOX, presumably due to structural differences to the avermectins. Higher mean *K*_OC_ in sediments than in soils were also documented by Chiou and Kile [70]. For a larger sample size, they described that for carbon tetrachloride and 1,2-dichlorobenzene mean sediment *K*_OC_ were about 1.7 times higher than soil *K*_OC_. Adding to that, we report sediment *K*_OC_ to soil *K*_OC_ ratios of 1.8 for ABA, 1.2 for DOR, 2.4 for IVM and 1.4 for MOX when comparing five selected sediments and 13 selected soils. Higher sediment than soil *K*_OC_ with a factor of about 1.9 was also shown for the antiparasitic drug albendazole by Mutavdžić Pavlović et al. [86]. Chiou and Kile [70] reported that during sedimentation, organic components fractionate and polar components dissolve over time, leaving behind hydrophobic components in the bed sediment.

Change of organic matter composition during sedimentation is known to affect sorption especially for nonionic compounds [73] and could be relevant for the examined antiparasitics. Their strong sorption in sediments is worrisome for inhabitants of these ecosystems, exemplarily shown by chronic effects of IVM on benthic invertebrates [45]. Adverse effects on sediment-dwelling non-target organisms must especially be considered when avermectins are applied in aquaculture [53]; an industry directly burdening aquatic ecosystems with VMPs [9] which may then pass into sediments. Davies et al. [34] expected risks for polychaetes living below or around fish cages and an IVM half-life in marine sediment  >  100 days. Prasse et al. [55] reported a comparable timeframe and documented high persistence of IVM in a simulated sediment/water system (DT_50_  =  127 days) driven by strong sorption in the sediment. Mesocosm experiments by Roberts et al. [87] with trout farm effluents showed moderate toxicity to benthic macroinvertebrates and no sensitive taxa were found in the receiving stream. The study, however, was performed unrelated to the use of pharmaceuticals. However, IVM is indicated to be highly persistent in sediments [44] and to possibly accumulate in aquatic organisms [47, 88]. This further encourages thorough, regulated exposure and risk assessments for hyporheic and benthic taxa. Sediment classification [64] and organic matter composition may also be relevant variables to predict *K*_D_ data.

### ***Relationship between K***_***OC***_*** and K***_***OW***_

The *K*_OC_ and *K*_OW_ of a substance are inextricably linked since both serve the concept that OC and 1-octanol act as hydrophobic counterparts to a chemical [89]. The *K*_OW_ is also the most frequently used indicator of hydrophobicity of a chemical and an essential parameter in toxicology and environmental sciences [90]. Over time, different concepts were developed to predict the sorption of organic chemicals in soils based on molecular properties. We ventured to predict the log *K*_OW_ of the studied antiparasitics if the RTO log *K*_OC_ were the only available variable. For this, we applied well-known concepts [68, 91–93] which aim to quantify the relationship between log *K*_OC_ and log *K*_OW_ based on log *K*_OW_ data. These predictions are depicted in Table 4. While it is apparent that conditions and limitations apply to these concepts, our decent set of log *K*_OC_ data should allow for an estimate of the antiparasitics’ hydrophobicity when expressed as log *K*_OW_. However, these estimations must not be overstated. A *K*_OW_ is easier to obtain experimentally than performing complex sorption batch studies. Hence, applying the slow-stirring method from OECD guideline 123 [35] would yield more accurate log *K*_OW_ data for the studied VMPs. The log *K*_OW_ of 5.6 (± 0.3) for IVM [13] defined this way could thus validate our own results. Estimates based on Gerstl [91] and Sabljić et al. [92] come closest to this value. This indicates a possible correlation which could also apply to the other three antiparasitics, especially when using log *K*_OC_ from sediment studies.

**Table 4:** Estimations for log *K*_OW_ of the investigated antiparasitics based on RTO log *K*_OC_ reported in this work. Compiled *K*_OC_–*K*_OW_ correlations are sorted chronologically

Substance in soils	Estimated log *K*_OW_ calculated from reported *K*_OC_–*K*_OW_ correlations			
	Karickhoff [68]^a^	Gerstl [91]^b^	Sabljić et al. [92]^c^	Baker [93]^d^
ABA	4.02	4.37	4.36	3.92
DOR	4.32	4.81	4.73	4.25
IVM	4.52	5.09	4.96	4.46
MOX	5.14	6.00	5.73	5.15
Substance in sediments				
ABA	4.42	4.96	4.85	4.36
DOR	4.53	5.11	4.98	4.47
IVM	5.01	5.81	5.57	5.00
MOX	5.38	6.34	6.01	5.40

^a^Original equation: $\log K_{{{\text{oc}}}}  = 0.989*\log K_{{{\text{ow}}}}  - 0.346$ (for hydrophobic chemicals)

^b^Original equation: $\log K_{{{\text{oc}}}}  = 0.679*\log K_{{{\text{ow}}}}  + 0.663$ (for non-specific chemicals)

^c^Original equation: $\log K_{{{\text{oc}}}}  = 0.81*\log K_{{{\text{ow}}}}  + 0.10$ (for predominantly hydrophobic chemicals)

^d^Original equation: $\log K_{{{\text{oc}}}}  = 0.903*\log K_{{{\text{ow}}}}  + 0.094$ (for non-specific chemicals)

The log *K*_OW_ is a hydrophobicity indicator linked to a molecule itself and it is immaterial whether said molecule would be released into soil, sediment, or other parts of the environment. Thus, an implied distinction between a log *K*_OW_ based on either soil or sediment sorption coefficients remains theoretical. Still, with the derived log *K*_OC_ data, all four substances displayed a log *K*_OW_  >  4 except for ABA in soils if calculated according to Baker [93]. This may indicate that in regulatory terms all drugs could carry a potential for bioaccumulation to occur in the environment [12] with IVM and MOX giving the biggest cause for concern in this regard. Then again, Tolls [11] described that the prediction from log *K*_OW_ could underestimate the log *K*_OC_. A reverse estimate based solely on sorption coefficients could therefore overestimate the log *K*_OW_. However, Tolls [11] also concluded that for large hydrophobic molecules such as avermectins log *K*_OC_ predictions would not deviate to a great extent which bolsters our predictions. The use of these estimations is to provide a general indication of hydrophobicity based on a common dataset of *K*_OC_ for all four substances.

Although more sophisticated approaches such as quantitative structure‐activity relationships can be employed, *K*_OC_ to *K*_OW_ correlations can be useful if transparent and verifiable *K*_OW_ data are not available. Benefits are conceivable since the *K*_OW_ is also an important parameter for environmental risk assessments. Prichard et al. [15] provided a consistent dataset of *K*_OW_ estimations and used atomic parameters to calculate the following order of coefficients (log *K*_OW_): EPR (4.4), IVM (4.8), ABA (5.3), DOR (5.6), MOX (6.0), and selamectin (6.3). Fittingly, selamectin was also assessed by Römbke et al. [13] with the slow-stirring method to indicate a log *K*_OW_ of 6.0 (± 0.7). Meanwhile, risk assessments for VMPs rely on robust data. Dissipation of macrocyclic lactone antiparasitics varies depending on climate and field conditions [20, 66, 94] and a harmonized dataset on experimental *K*_OW_ and their environmental fate properties would be admirable.

A limitation of sorption studies with pharmaceutical compounds is the transferability to the environmental reality. The *K*_OC_ concept does not account for organic matter composition and may misinterpret substance behavior at particular locations, especially in sediments. If enough data is available, a multiple linear regression with all soil/sediment properties is always advisable. Also, while for IVM low metabolization has been described in animal species [14], human metabolism of IVM could be more pronounced [81]. Transformation products of varying size and polarity could hypothetically demonstrate different sorption behavior in soils and sediments. Investigating the abundance and fate of antiparasitic metabolites after excretion is thus a logical future task. In light of the upcoming European veterinary regulation [Regulation (EU) 2019/6] steadfast assessments will gain in importance [39]. Our estimations of log *K*_OW_ based on log *K*_OC_ highlight the possible *K*_OW_ discrepancies and a precarious aspect of regulatory decision-making: while data may appear insufficient, they may be the only data available.

## Conclusions

The investigated antiparasitics show strong sorption to the organic matter of soils and also sediments. Sorption strength in general (as *K*_D_) and normalized to organic carbon (as *K*_OC_) is characterized by the order: ABA  <  DOR  <  IVM  <<  MOX. Exemplary desorption from soils indicates mostly irreversible sorption processes and follows the same rationale with MOX showing the lowest transfer back into the liquid phase. The applied SPE-HPLC method with fluorescence detection is suitable for reliable quantification of all four analytes at once.

The consequent use of linear modeling with constrained intercepts allows to derive transparent and comparable sorption coefficients and facilitates future referral to our dataset. A variety of *K*_OW_ estimates urges to re-assess this important regulatory parameter with the appropriate technique. While for IVM and MOX our findings suggest the need to examine potential aquatic or terrestrial bioaccumulation, the medical and economic benefits of all four pharmaceuticals must not be denied. It is thus desirable to elaborate on their environmental fate and also include sediment-dwelling organisms in frameworks for toxicity testing. In perspective, risk mitigation measures for macrocyclic lactones should be improved to make antiparasitics a luminous example for the sustainable use of veterinary pharmaceuticals.

## Supplementary Information

**Additional file 1: Table S1.** Analyte recovery (%) when subjected to the presented SPE procedure compared to directly measured standards. **Table S2.** Output (OriginPro 2020b) of the multiple linear regression with transformed K_D_ values and soil properties. **Table S3.** Supplemental data for Fig. 2. Individual soil K_D_ values (mL/g). **Table S4.** Supplemental data for Fig. 3. Individual sediment K_D_ values (mL/g). **Figure S1.** Chromatogram of standard solution with all 4 analytes (Abamectin, ABA; Doramectin, DOR; Ivermectin, IVM; Moxidectin, MOX). **Figure S2.** Chromatogram of the extracted aqueous soil solution with all 4 analytes (Abamectin, ABA; Doramectin, DOR; Ivermectin, IVM; Moxidectin, MOX).

## Data Availability

All relevant data and material are included in this published article and its supplementary information (SI). Other data and calculation tools for this research are available upon reasonable request from the authors A. P. Heinrich and R-A. Düring.

## Abbreviations

ABA: Abamectin
ACN: Acetonitrile
DOR: Doramectin
EMA: European Medicines Agency
EPR: Eprinomectin
EU: European Union
*f*_OC_: Organic carbon fraction of the soil/sediment
HLNUG: Hessian Agency for Nature Conservation, Environment and Geology
IVM: Ivermectin
*K*_D_: Distribution coefficient
*K*_OC_: Organic carbon–water partition coefficient
*K*_OW_: Octanol–water partition coefficient
LOD: Limit of detection
LOQ: Limit of quantification
MOX: Moxidectin
OC: Organic carbon
OECD: Organisation for Economic Co-operation and Development
OLS: Ordinary least squares regression
%OC: Weight percentage of soil/sediment organic carbon
RTO: Regression through the origin
SE: Standard error
VMPs: Veterinary medicinal products
