# Supplementary material for: Sorption of selected antiparasitics in soils and sediments
Source: Environ Sci Eur. 2021 Jul 2;33(1):77. doi: 10.1186/s12302-021-00513-y (PMC8253237; doi:10.1186/s12302-021-00513-y)
Supplement: Supplementary file 1 — Additional file 1: Table S1. Analyte recovery (%) when subjected to the presented SPE procedure compared to directly measured standards. Table S2. Output (OriginPro 2020b) of the multiple linear regression with transformed KD values and soil properties. Table S3. Supplemental data for Fig. 2. Individual soil KD values (mL/g). Table S4. Supplemental data for Fig. 3. Individual sediment KD values (mL/g). Figure S1. Chromatogram of standard solution with all 4 analytes (Abamectin, ABA; Doramectin, DOR; Ivermectin, IVM; Moxidectin, MOX). Figure S2. Chromatogram of the extracted aqueous soil solution with all 4 analytes (Abamectin, ABA; Doramectin, DOR; Ivermectin, IVM; Moxidectin, MOX). [file 12302_2021_513_MOESM1_ESM.pdf]

Additional File 1

Sorption of Selected Antiparasitics in Soils and Sediments

Heinrich, Andre Patrick[1]; Zöltzer, Timm[1]; Böhm, Leonard[1]; Wohde, Manuel[1]; Jaddoudi, Sara[2]; El Maataoui, Yassine[2]; Dahchour, Abdelmalek[3]; Düring, Rolf-Alexander[1]

[1] Institute of Soil Science and Soil Conservation, Research Center for Biosystems, Land Use and Nutrition (iFZ), Justus Liebig University Giessen, Giessen, Germany

[2] Laboratory of Materials, Nanotechnology and Environment (LMNE), Faculty of Sciences, Mohammed V University in Rabat, Av Ibn Battouta Agdal, BP1014, Rabat, Morocco

[3] Département Des Sciences Fondamentales et Appliquées Institut Agronomique et Véterinaire Hassan II Rabat, Morocco

I – The solutions of the calibration curve were not prepared using the solid phase extraction (SPE) procedure. To address this, we performed an additional experiment with n = 6 replicates each to compare standard solution measured directly and standard solution added to 0.01 mol/L $CaCl_2$ which then underwent identical sample processing as described in the manuscript (including SPE and filtration). These recoveries are listed below in Table S1.

Table S1: Analyte recovery (%) when subjected to the presented SPE procedure compared to directly measured standards.

	Abamectin (%)	Doramectin (%)	Ivermectin (%)	Moxidectin (%)
Mean (n = 6)	96.8	96.1	95.8	93.0
Standard deviation	1.8	1.4	2.1	1.7

II – The output (OriginPro 2020b) of the multiple linear regression (see "Results and Discussion - Sorption in soils") is shown below in table S2. The comparison with soil properties from table 1 in the manuscript is as follows (α = 5 %):

Table S2: Output (OriginPro 2020b) of the multiple linear regression with transformed K_D values and soil properties.

	ABA K_D	DOR K_D	IVM K_D	MOX K_D
n =	20	20	20	14
	Prob>|t|	Prob>|t|	Prob>|t|	Prob>|t|
**%OC**	0.008	0.013	0.009	0.215
**C/N**	0.046	0.042	0.114	0.372
**pH**	0.029	0.021	0.051	0.120
**CEC**	0.180	0.209	0.315	0.250
**Sand**	0.709	0.917	0.876	0.252
**Silt**	0.708	0.918	0.876	0.253
**Clay**	0.710	0.915	0.875	0.253

III – Characteristic HPLC-fluorescence chromatograms of standard solution with all 4 analytes (Abamectin, ABA; Doramectin, DOR; Ivermectin, IVM; Moxidectin, MOX) and of the extracted aqueous soil solution are depicted in figures S1 and S2.

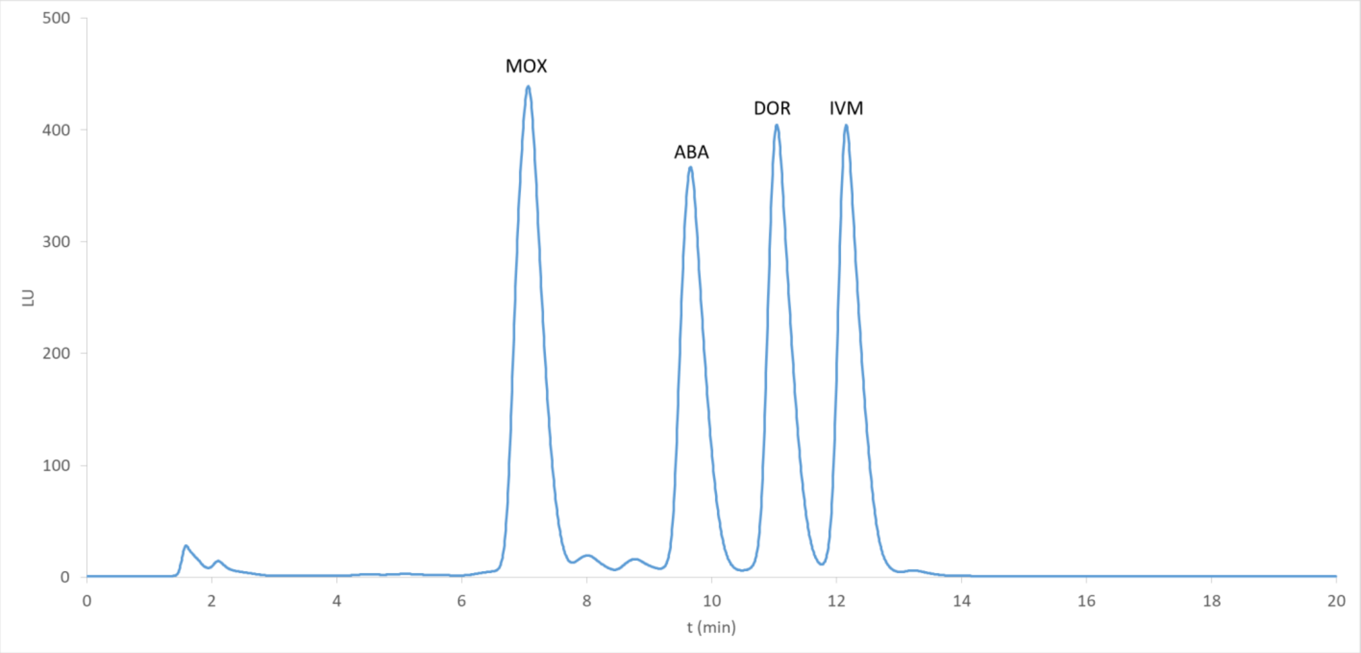

Figure S1: Chromatogram of standard solution with all 4 analytes (Abamectin, ABA; Doramectin, DOR; Ivermectin, IVM; Moxidectin, MOX).

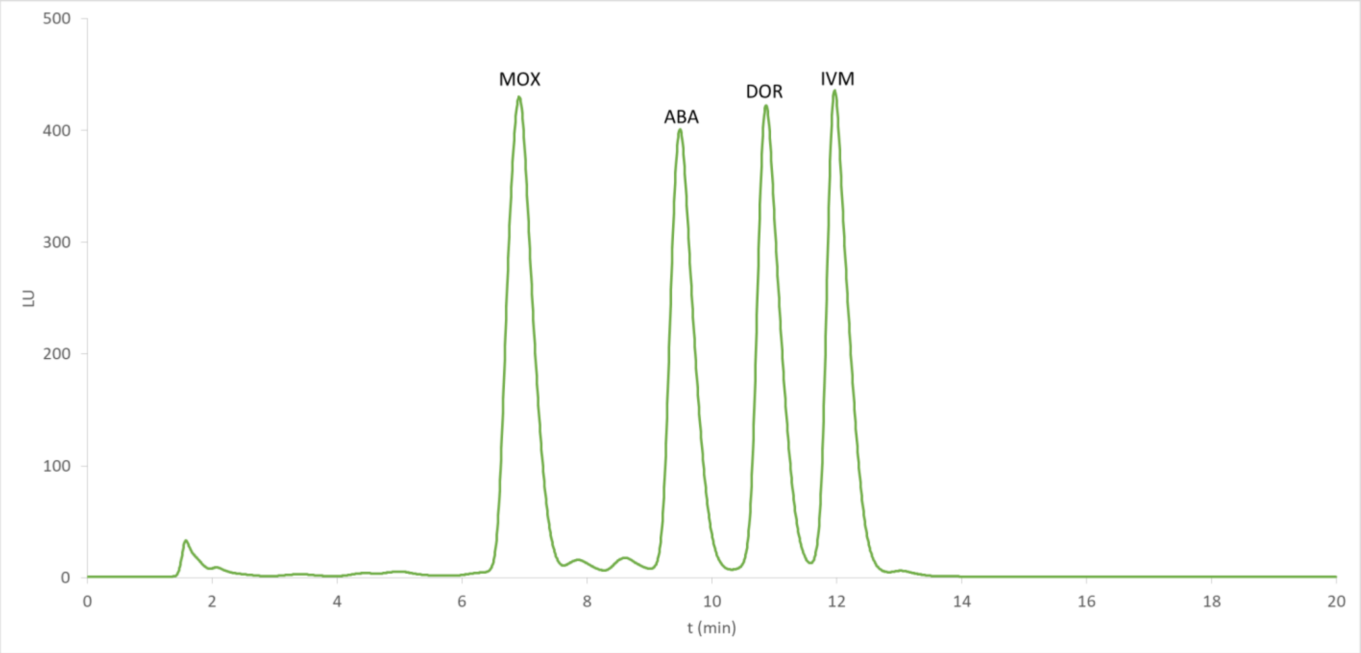

Figure S2: Chromatogram of the extracted aqueous soil solution with all 4 analytes (Abamectin, ABA; Doramectin, DOR; Ivermectin, IVM; Moxidectin, MOX).

IV – Supplemental data for manuscript figures 2 and 3. Individual Soil K_D values (mL/g) for soils (Table S3) and sediments (Table S4).

Table S3: Supplemental data for figure 2. Individual soil K_D values (mL/g).

Soil	f_{OC}[a]	ABA K_D	DOR K_D	IVM K_D	MOX K_D
DE01	0.001	52.6	84.2	81.7	n/d
DE06	0.001	107.3	163.5	176.9	n/d
DE04	0.001	52.5	140.1	75.0	n/d
DE05	0.002	46.9	71.5	79.8	n/d
DE07	0.003	84.3	168.3	188.1	227.6
DE03	0.007	37.8	70.9	76.2	n/d
DE10	0.008	47.9	92.0	136.6	276.5
DE16	0.009	48.7	94.8	101.3	166.3
DE08	0.010	130.5	367.4	251.5	535.8
MA03	0.013	48.5	63.2	143.9	580.1
DE09	0.018	73.8	152.9	230.0	985.1
MA02	0.019	66.8	84.6	216.9	994.5
MA01	0.021	55.5	71.7	185.3	756.0
DE11	0.027	92.2	181.4	271.8	1146.2
DE12	0.031	134.3	270.7	424.2	1915.2
DE13	0.036	154.8	316.1	492.2	1623.6
DE14	0.039	172.8	362.9	561.6	2249.2
DE17	0.047	210.8	427.6	641.5	3123.2
DE02	0.059	93.4	202.0	259.8	n/d
DE15	0.060	127.0	259.2	386.7	2193.7

[a] f_{OC} is the organic carbon fraction of the soil

n/d = not determined

Table S4: Supplemental data for figure 3. Individual sediment KD values (mL/g).

Sediment	f_{OC}[a]	ABA K_D	DOR K_D	IVM K_D	MOX K_D
MA06	0.004	296.1	375.8	915.4	783.2
MA04	0.004	21.5	35.2	52.6	87.2
MA09	0.006	36.3	47.5	109.1	191.8
MA05	0.012	143.8	187.1	482.3	625.4
MA08	0.014	145.4	175.5	526.4	1076.2
MA07	0.016	181.7	237.6	800.7	2325.5

[a] f_{OC} is the organic carbon fraction of the sediment
